# Knowledge on voluntary medical male circumcision in a low uptake setting in northern Uganda

**DOI:** 10.1186/s12889-018-6158-2

**Published:** 2018-11-20

**Authors:** Barbara Marjorie Nanteza, David Serwadda, Edward Nelson Kankaka, Grace Bua Mongo, Ronald Gray, Frederick Edward Makumbi

**Affiliations:** 1 Department of epidemiology and biostatistics, Makerere University School of Public Health, Kampala, Uganda; 2 Uganda Ministry of Health, AIDS Control Program- National Male Circumcision office, Kampala, Uganda; 3 Rakai Health Sciences Program, Department of Grants, Training & Science, Rakai, Uganda; 4 Johns Hopkins Bloomberg School of Public health, Department of Epidemiology, Baltimore, MD USA

**Keywords:** Knowledge, Misconception, Circumcision, VMMC, Sexual function, Uganda, Sub-Saharan Africa

## Abstract

**Background:**

Free VMMC services have been available in Uganda since 2010. However, uptake in Northern Uganda remains disproportionately low. We aimed to determine if this is due to men’s insufficient knowledge on VMMC, and if women’s knowledge on VMMC has any association with VMMC status of their male sexual partners.

**Methods:**

In this cross sectional study, participants were asked their circumcision status (or that of their male sexual partner for female respondents) and presented with 14 questions on VMMC benefits, procedure, risk, and misconceptions. Chi square tests or fisher exact tests were used to compare circumcision prevalence among those who gave correct responses versus those who failed to and if *p* < 0.05, the comparison groups were balanced with propensity score weights in modified poisson models to estimate prevalence ratios, PR.

**Results:**

A total of 396 men and 50 women were included in the analyses. Circumcision was 42% less prevalent among males who failed to reject the misconception that VMMC reduces sexual performance (PR = 0.58, 95% CI 0.38–0.89, *p* = 0.012), and less prevalent among male sexual partners of females who failed to reject the same misconception (PR = 0.22, 95% CI = 0.07–0.76, *p* = 0.016). Circumcision was also 35% less prevalent among male respondents who failed to reject the misconception that VMMC increases a man’s desire for more sexual partners i.e. promiscuity (PR = 0.65, 95% CI = 0.46–0.92, *p* = 0.014).

**Conclusion:**

Misconceptions regarding change in sexual drive or performance were associated with circumcision status in this population, while knowledge of VMMC benefits, risks and procedure was not.

**Electronic supplementary material:**

The online version of this article (10.1186/s12889-018-6158-2) contains supplementary material, which is available to authorized users.

## Background

Three randomized trials demonstrated that Voluntary Medical Male Circumcision (VMMC) leads to a 50–60% relative risk reduction of female to male HIV transmission [1–3]. The World Health Organization (WHO) and UNAIDS consequently recommended scale up of VMMC for HIV prevention settings as an additional important strategy for prevention of heterosexually acquired HIV infection in men [4]. Uganda in particular needs to circumcise 6.9 million men aged 10–49 by 2020 in order to halve HIV transmission compared to 2010. However, the annual number of circumcisions peaked in 2014, only 2.5 million men (59.5%) had been circumcised by 2016, and the initial regional disparities in circumcision levels persisted [5, 6]. In particular, some high HIV prevalence regions like Northern Uganda (7.2% HIV prevalence versus 6.2% national average) remain with disproportionately low circumcision levels [7].

In order to accelerate program progress, intensifying efforts such as targeted messaging to specific regions and age groups has been proposed [8, 9]. The Uganda National communication strategy for circumcision in HIV prevention specifies key messages to different target audiences [10]. The strategy guides communication to facilitate recruitment of eligible uncircumcised males in communities, and its used to develop Information Education & Communication /Behaviour Change Communication (IEC/BCC) materials which are simplified, illustrated, and translated to local languages to suit the setting. Although this strategy has been in effect since 2010 and free VMMC services are available, it is still unclear whether the low VMMC uptake in such settings is a knowledge problem. This study sought to determine the relationship between men’s knowledge on VMMC and their circumcision status, and if women’s knowledge on VMMC has any association with VMMC status of their male sexual partners.

## Methods

### Study setting

The study was conducted in Gulu district in mid-northern Uganda. This region was the centre of a 20-year civil war, still has a high HIV prevalence above the national average (7.2 versus 6.2), and has a persistently low VMMC coverage [7]. Gulu district has an urban county (Gulu municipality) and two rural counties (Achwa and Omoro). VMMC services are available free-of-charge using funding from development partners, and are provided mostly through scheduled community outreaches, and also routinely at four health facilities - two in the urban county and two in the rural counties.

### Study design and participants

In this cross-sectional study, we recruited consenting adults aged 18–49 years living in a household within Gulu district for more than 6 months by December 2015, and were within a 4 km radius from a health facility with free circumcision services. Men were the primary interest group, but women with male sexual partners were also included to determine if their knowledge had any association with circumcision status of their male sexual partners. Initially, service providers were included but were interviewed and found to have universal knowledge.

### Study procedures

Systematic sampling was used to select households in each of the three counties of Gulu district (2 rural and 1 urban) using lists from a recent mosquito net distribution campaign. Eligible residents were ranked by age in descending order, and potential participants were randomly selected using a KISH grid [11]. These were then contacted for a possible interview, with two additional attempts for respondents who were not initially reached. A semi-structured questionnaire which was developed for this study (Additional file 1), was pre-tested at a non-study site, was used to collect cross-sectional data on sociodemographic characteristics, men’s circumcision status or circumcision status of the male sexual partner for female respondents (irrespective of whether the circumcision was medical or non-medical), and knowledge on VMMC. Sociodemographic characteristics included age, location, marital status, tribe, religion, education level, and occupation. The questions for knowledge on VMMC were selected from the National communication strategy [10], administered in a respondent’s preferred language, and included priority questions on VMMC benefits (*n* = 4 questions), risks (*n* = 1), procedure (*n* = 3), and misconceptions (*n* = 6). Misconceptions ought to be rejected, while facts on benefits, risks, and procedure ought to be accepted.

### Statistical analysis

Frequencies and percentages were used to describe categorical sociodemographic characteristics of the participants, and means(SD) and medians(IQR) were used for age. Correct acceptance of a fact or correct rejection of a misconception was scored 1, otherwise 0. Chi square tests or fisher exact tests were used to determine association between responses to each of the 14 selected/priority knowledge questions and circumcision status, the outcome of interest. For questions significantly associated with circumcision status, propensity score weighting (inverse probability of treatment weights) was then applied to balance those who gave correct responses and those who did not with respect to sociodemographic characteristics as potential confounders. Propensity scores were preferred over conventional regression modelling since they are much less vulnerable to model misspecification when dealing with measured confounders [12, 13] The balanced groups were then compared using modified poisson models to obtain prevalence ratios (PR), excluding subjects outside the common range of propensity scores. Prevalence Ratios (PR) were used because data were cross-sectional, they can convey strength of association between exposure and outcome, and are more conservative in magnitude than Prevalence Odds Ratios (POR) when the outcome is relatively common i.e. > 10% [14, 15], and have been used elsewhere in cross-sectional studies relating knowledge to circumcision [16, 17]. Modified poisson regression with robust variance can be used to estimate PR for cross-sectional data when log-binomial models fail to converge, which occurs quite often [15, 18]. To check potential influence of missing data on results, participants with missing responses to the selected knowledge questions were compared to those with complete responses using chi square tests or fisher exact tests as appropriate. Male and female respondents were analysed separately but results are presented side by side for comparison. STATA 14.2 was used for analysis [19].

## Results

A total of 488 participants participated in the study; 428 were male and 60 were female. Circumcision prevalence was 30.6% among male respondents, and 26.7% among male sexual partners of female respondents. Complete response to all selected questions for VMMC knowledge was 92.5% (*n* = 396) among male respondents, and 83.3% (*n* = 50) among female respondents. We subsequently analysed respondents with complete responses, since those with incomplete responses were not different from those with complete responses with respect to sociodemographic characteristics and VMMC status, Additional file 2: Table S1.

### Sociodemographic characteristics

The median age of male respondents was 25 years (IQR 22–29), and most were of the Acholi tribe (86.6%), 68.9% were catholic, and 72.7% had a secondary school education or higher. The Acholi tribe are a traditionally non-circumcising tribe. Location (rural or urban), tribe, education level, and occupation were associated with circumcision status of male respondents. In contrast, the median age of female respondents was 26.5 years (IQR 23–32), most were also of the Acholi tribe (84%), 64% were catholic, but less than half (46%) had a secondary school education or higher. Female respondents’ sociodemographic characteristics had no association with circumcision status of their male sexual partners, Table 1.

**Table 1:** Sociodemographic characteristics of male and female respondents

	Male respondents (*N* = 396)				Female respondents (*N* = 50)			
	All (*N* = 396)	Uncircumcised (*n* = 272)	Circumcised (*n* = 124)	p	All (*N* = 50)	Sexual partner uncircumcised (*n* = 34)	Sexual partner circumcised (*n* = 16)	p
Age								
Mean(SD)	26.5(6.8)	26.7(6.8)	26.1(6.8)	0.390	28.4(7.8)	28.3(8.1)	28.6(7.4)	0.890
Median(IQR)	25(22–29)	25(22–30)	24(22–28)	0.345	26.5(23–32)	26.5(23–32)	26(23.5–33.5)	0.771
Location, n(%)								
Rural	178(45.0)	133(48.9)	45(36.3)	0.019	25(50.0)	16(47.1)	9(56.3)	0.544
Urban	218(55.0)	39(51.1)	79(63.7)		25(50.0)	18(52.9)	7(43.7)	
Marital status, n(%)								
Married/consensual	210(53.0)	53(56.3)	7(46.0)	0.057	32(64.0)	22(64.7)	10(62.5)	0.880
Unmarried	186(47.0)	19(43.8)	67(54.0)		18(36.0)	12(35.3)	6(37.5)	
Tribe, n(%)								
Acholi	343(86.6)	244(89.7)	99(79.8)	0.003	42(84.0)	30(88.2)	12(75.0)	0.331
Langi	39(9.9)	24(8.8)	5(12.1)		2(4.0)	1(2.9)	1(6.3)	
Other	14(3.5)	4(1.5)	10(8.1)		6(12.0)	3(8.8)	3(18.8)	
Religion, n(%)								
Catholic	273(68.9)	191(70.2)	82(66.1)	0.357	32(64.0)	23(67.7)	(56.3)	0.466
Protestant	76(19.2)	53(19.5)	23(18.6)		13(26.0)	9(26.5)	4(25.0)	
Other	47(11.9)	28(10.3)	19(15.3)		5(10.0)	2(5.9)	3(18.8)	
Education, n(%)								
None to primary	108(27.3)	91(33.5)	17(13.7)	< 0.001	27(54.0)	21(61.8)	6(37.5)	0.118
Secondary	185(46.7)	123(45.2)	62(50.0)		17(34.0)	11(32.3)	6(37.5)	
Tertiary/university	103(26.0)	58(21.3)	45(36.3)		6(12.0)	2(5.9)	4(25.0)	
Occupation, n(%)								
Farming	102(25.8)	80(29.4)	22(17.7)	0.018	12(24.0)	7(20.6)	5(31.3)	0.139
Trading	82(20.7)	60(22.1)	22(17.7)		21(42.0)	18(52.9)	3(18.8)	
Student	62(15.7)	8(14.0)	24(19.4)		3(6.0)	1(2.9)	2(12.5)	
Other	104(26.3)	61(22.4)	43(34.7)		9(18.0)	5(14.7)	4(25.0)	
Unemployed	46(11.6)	33(12.1)	13(10.5)		5(10.0)	3(8.8)	2(12.5)	

### The association between men’s knowledge on VMMC and their circumcision status

Bivariate analyses (Table 2) showed that majority of men did not know that VMMC reduces cervical cancer risk for female partner (92.7% failed to accept), and most erroneously thought VMMC significantly reduces risk of HIV transmission to female partner (75.5% failed to reject). However, these were not associated with circumcision status (*p* > 0.05). Instead, the misconception that VMMC reduces sexual performance was strongly associated with circumcision status (*p* = 0.001). Also associated with circumcision status was knowledge whether men normally bleed after VMMC (*p* = 0.006), the misconception that men usually desire more sexual partners after VMMC i.e. that VMMC increases promiscuity (*p* = 0.016), and knowledge whether VMMC differs from traditional circumcision (*p* = 0.042).

**Table 2:** Association between knowledge on VMMC and VMMC status

	Male respondents				Female respondents			
	All (*N* = 396)	Uncircumcised (*n* = 272)	Circumcised (*n* = 124)	p	All (*N* = 50)	Sexual partner uncircumcised (*n* = 34)	Sexual partner circumcised (*n* = 16)	p
VMMC reduces a man’s risk of acquiring HIV, n(%)								
Accept	362(91.4)	249(91.5)	113(91.1)	0.891	47(94.0)	32(94.1)	5(93.8)	1.000
Fail to accept	34(8.6)	23(8.5)	11(8.9)		3(6.0)	2(5.9)	1(6.3)	
VMMC improves genital hygiene, n(%)								
Accept	377(95.2)	257(94.5)	120(96.8)	0.449	49(98.0)	33(97.1)	16(100.0)	1.000
Fail to accept	19(4.8)	15(5.5)	4(3.2)		1(2.0)	1(2.9)	0(0.0)	
VMMC reduces risk of STIs, n(%)								
Accept	317(80.0)	215(79.0)	102(82.3)	0.458	35(70.0)	23(67.7)	2(75.0)	0.746
Fail to accept	79(20.0)	57(21.0)	22(22.7)		15(30.0)	11(32.4)	4(25.0)	
VMMC reduces cervical cancer risk for female partner, n(%)								
Accept	29(7.3)	22(8.1)	7(5.6)	0.387	7(14.0)	3(8.8)	4(25.0)	0.190
Fail to accept	367(92.7)	250(91.9)	117(94.4)		43(86.0)	31(91.2)	12(75.0)	
VMMC involves removal of the foreskin, n(%)								
Accept	389(98.2)	266(97.8)	123(99.2)	0.442	50(100.0)	34(100.0)	6(100.0)	–
Fail to accept	7(1.8)	6(2.2)	1(0.8)		0(0.0)	0(0.0)	0(0.0)	
VMMC differs from traditional circumcision, n(%)								
Accept	300(75.8)	198(72.8)	102(82.3)	0.042	25(50.0)	17(50.0)	8(50.0)	1.000
Fail to accept	96(24.2)	74(27.2)	22(17.7)		25(50.0)	17(50.0)	8(50.0)	
An injection is given for pain control before VMMC, n(%)								
Accept	378(95.5)	257(94.5)	121(97.6)	0.203	40(80.0)	25(73.5)	15(93.7)	0.138
Fail to accept	18(4.5)	15(5.5)	3(2.4)		10(20.0)	9(26.5)	1(6.3)	
Men normally bleed after VMMC, n(%)								
Reject	301(76.0)	196(72.1)	105(84.7)	0.006	36(72.0)	23(67.7)	3(81.3)	0.501
Fail to reject	95(24.0)	76(27.9)	19(15.3)		14(28.0)	11(32.4)	3(18.8)	
Tubes that carry sperm are cut during VMMC, n(%)								
Reject	360(90.9)	45(90.1)	115(92.7)	0.392	42(84.0)	7(79.4)	15(93.7)	0.409
Fail to reject	36(9.1)	27(9.9)	9(7.3)		8(16.0)	7(20.6)	1(6.3)	
VMMC can make a man immune to acquiring HIV, n(%)								
Reject	359(90.7)	48(91.2)	111(89.5)	0.582	40(80.0)	27(79.4)	13(81.2)	1.000
Fail to reject	37(9.3)	24(8.8)	13(10.5)		10(20.0)	7(20.6)	3(18.8)	
VMMC makes a man a muslim, n(%)								
Reject	347(87.6)	234(86.0)	113(91.1)	0.153	43(86.0)	29(85.3)	14(87.5)	1.000
Fail to reject	49(12.4)	38(14.0)	11(8.9)		7(14.0)	5(14.7)	2(12.5)	
VMMC reduces risk of HIV transmission to female partner, n(%)								
Reject	97(24.5)	68(25.0)	29(23.4)	0.729	8(16.0)	4(11.8)	4(25.0)	0.249
Fail to reject	299(75.5)	205(75.0)	95(76.6)		42(84.0)	30(88.2)	12(75.0)	
Men usually desire more sexual partners after VMMC, n(%)								
Reject	230(58.1)	47(45.0)	83(66.9)	0.016	25(50.0)	5(44.1)	10(62.5)	0.225
Fail to reject	66(41.9)	25(46.0)	41(33.1)		25(50.0)	19(55.9)	6(37.5)	
VMMC reduces sexual performance, n(%)								
Reject	284(71.7)	181(66.5)	103(83.1)	0.001	25(50.0)	13(38.2)	12(75.0)	0.032
Fail to reject	12(28.3)	91(33.5)	21(16.9)		25(50.0)	21(61.8)	4(25.0)	

After applying propensity weights in poisson models to balance the comparison groups (Additional file 3: Table S2), male respondents who failed to reject the misconception that VMMC reduces sexual performance had 42% lower prevalence of circumcision (PR = 0.58, 95% CI 0.38–0.89, *p* = 0.012). Also, men who failed to reject the misconception that VMMC increases a man’s desire for more sexual partners had 35% lower prevalence of circumcision (PR = 0.65, 95% CI = 0.46–0.92, *p* = 0.014), Table 3.

**Table 3:** Association of knowledge with VMMC status, using propensity score weighting, excluding subjects outside the common support

		Crude PR (95% CI)	*p*-value	Weighted.PR (95% CI) ^a^	*p*-value
Male respondents	VMMC differs from traditional circumcision				
	Fail to accept vs Accept	0.67(0.45–1.01)	0.053	0.77(0.51–1.15)	0.204
	Men normally bleed after VMMC				
	Fail to reject vs Reject	0.57(0.37–0.88)	0.011	0.67(0.41–1.08)	0.103
	Men usually desire more sexual partners after VMMC				
	Fail to reject vs Reject	0.68(0.50–0.94)	0.019	0.65 (0.46–0.92)	0.014*
	VMMC reduces sexual performance				
	Fail to reject vs Reject	0.52(0.34–0.78)	0.002	0.58(0.38–0.89)	0.012*
Female respondents	VMMC reduces (men’s)sexual performance				
	Fail to reject vs Reject	0.33(0.12–0.90)	0.031	0.22(0.07–0.76)	0.016*

^a^ Prevalence ratio from poisson model with propensity score weights, excluding respondents outside the common support

* Association with circumcision (*p* < 0.05)

### Association between women’s knowledge on VMMC and the circumcision status of their male sexual partners

Bivariate analyses (Table 2) for female respondents showed that majority of female respondents (91.2%) did not know that VMMC reduces their risk for cervical cancer, and most (88.2%) also erroneously thought VMMC reduces their risk of HIV acquisition from male sexual partners. However, these were not associated with circumcision status (*p* > 0.05). Instead, the misconception that VMMC reduces a man’s sexual performance was significantly associated with circumcision status of their male sexual partners (*p* = 0.032). After applying propensity weights in poisson models, male sexual partners of female respondents who failed to reject misconception that VMMC reduces sexual performance had significantly lower prevalence of circumcision, although the estimate was imprecise (PR = 0.22, 95% CI = 0.07–0.76, *p* = 0.016).

## Discussion

It is interesting to note that knowledge of VMMC benefits, risk, and procedure was not associated with circumcision status of male respondents, or circumcision status of male sexual partners of female respondents. Instead, concern of reduced sexual performance was strongly associated with circumcision status of male respondents, as well as circumcision status of male sexual partners of female respondents. A qualitative study revealed similar concerns among Swazi men [20], but did not evaluate association with actual circumcision status and did not include female respondents. To date, several systematic reviews and meta-analyses have shown no empirical evidence of reduced in sexual performance in circumcised men [21–24]. Similarly, the misconception that men desire more sexual partners after VMMC i.e. become more promiscuous was also independently associated with circumcision status of male respondents. This is probably a moral or religious concern, also with no empirical evidence to date [21, 23, 24]. These misconceptions regarding changes in sexuality following VMMC need to be effectively addressed in these communities, perhaps using novel approaches different from those in current use, and the novel approaches should ideally include female sexual partners whenever applicable and possible.

### Limitations

We could not establish whether the knowledge preceded circumcision given the cross-sectional design i.e. temporality between exposure and outcome, but the observed associations could plausibly explain the reluctance of men in this setting to get circumcised, and the reluctance of women to encourage their male sexual partners. Unmeasured confounders may explain away the observed association, but sensitivity analyses for these would also involve more untestable assumptions. Subsequent studies could establish temporality between messaging and subsequent circumcision; and also explore exposure to specific VMMC messages especially those addressing misconceptions and relationship with actual knowledge or attitudes to check whether messages are truly effective or not. A qualitative study in the same setting could complement our findings by further exploring cultural influences and other attitudes.

## Conclusion

Misconceptions regarding change in sexual drive or performance were associated with VMMC status in this population, while knowledge of VMMC benefits, risks and procedure was not.

## Additional files

Additional file 1: Questionnaire used to assess knowledge on HIV and VMMC in a low uptake setting in Northern Uganda. This questionnaire was developed from the Uganda National communication strategy for circumcision in HIV prevention. We prioritized questions on VMMC benefits (*n* = 4 [qn18, qn20, qn22, qn23]), risks (*n* = 1 [qn25]), procedure (*n* = 3 [qn7, qn11, qn14]), and misconceptions (*n* = 6 [qn16, qn17, qn21, qn24, qn34, qn36]). Socio-demographics and priority questions used for this paper are marked with an asterisk (*) in supplement 3. (DOC 129 kb)

Additional file 2: **Table S1.** Comparison of participants with complete responses versus those with incomplete responses to knowledge questions on VMMC. (DOC 45 kb)

Additional file 3: **Table S2.** Balancing of comparison groups with propensity score weights. Numbers are Standardized mean differences (SMD), and an absolute SMD < 0.1 is desirable for each observed covariate. Only questions with *p* < 0.05 in bivariate analyses (Table 1) are included. (DOC 50 kb)

## Abbreviations

AIDS: Acquired Immunodeficiency syndrome
BCC: Behavioral Communication Change
HIV: Human immunodeficiency Syndrome
IEC: Information Education and Communication
UNAIDS: United Nations Programme on HIV and AIDS
VMMC: Voluntary Medical Male Circumcision
WHO: World Health Organization
