# Supplementary material for: Knowledge on voluntary medical male circumcision in a low uptake setting in northern Uganda
Source: BMC Public Health. 2018 Nov 20;18:1278. doi: 10.1186/s12889-018-6158-2 (PMC6245765; doi:10.1186/s12889-018-6158-2)
Supplement: Supplementary file 1 — Questionnaire used to assess knowledge on HIV and VMMC in a low uptake setting in Northern Uganda. This questionnaire was developed from the Uganda National communication strategy for circumcision in HIV prevention. We prioritized questions on VMMC benefits (n = 4 [qn18, qn20, qn22, qn23]), risks (n = 1 [qn25]), procedure (n = 3 [qn7, qn11, qn14]), and misconceptions (n = 6 [qn16, qn17, qn21, qn24, qn34, qn36]). Socio-demographics and priority questions used for this paper are marked with an asterisk (*) in supplement 3. (DOC 129 kb) [file 12889_2018_6158_MOESM1_ESM.doc]

Preamble

The main purpose of this study is to assess knowledge of Ministry of Health-approved Voluntary Medical Male Circumcision (VMMC) messages. I will ask you questions about HIV infection, Voluntary Medical Male Circumcision (VMMC), and other interventions that prevent the spread of HIV. All information that you provide will be kept confidential and will not be shared with other persons with your identifiers.

dd/ mm/ yyyy

***Date of Interview**  _____/____/201_

***Participant id** _______________

**Interviewer’s Name** __________________________________

**SOCIO-DEMOGRAPHICS**

*Where is your home **located**?

Lalogi 01

Awach 02

Municipality 03

Sub-county

Parish ________________________ Village________________________

How old are you:

***Age** (years): * **Sex**:

Female-1 Male-2

Acholi -1 Langi-2 Ganda-3 Other, Specify _______­­­____4

*What is your **tribe?**

Catholic-1 Anglican-2, Born Again-3 SDA-4 Muslim-5

Other, Specify _______6

*What is your **religion?**

Have you ever **gone to school**? Y/N

None-1, Primary-2, Secondary-3, Tertiary /University-4

Other_specify__________5

*What is your highest **level of education**

attained:

Peasant-1, farming-2, Casual Laborer-3,

Shop Attendant – 4, Teacher-5 Business Man/woman-6, Health Care Worker -7 Civil Servant-8, student-9,

Bodaboda rider-10, market vendor – 11, unemployed -12

Other, Specify______ ________ 9

*What is your primary **occupation**?

(Occupation where you spend most of your

time on a typical day)

*Are you **circumcised**? (if female is your

Circumcised 1, Not circumcised – 2

Don’t Know - 3

Not applicable -4

spouse/partner/husband circumcised?)

Circumcision Status (respondent/spouse)

**HIV/AIDS AWARENESS, RISK, and OTHER PREVENTION (except VMMC)**

*(In this section, select 1 for a “Yes", 2 for a “No”, and DK for “Do not know”)*

I would like to ask you about HIV/AIDS, how it is spread and how it can be prevented:

Yes No DK

Qn1 Have you ever heard about HIV/AIDS? 1 2 3

Qn2 Can you get HIV by sharing food with an HIV infected person? 1 2 3

Qn3 Can you get HIV by having unprotected sex with an infected

person or someone whose HIV status you do not know? 1 2 3

Qn4 Drugs called ARVs are given to people living with HIV:

can ARVs cure HIV/AIDS? 1 2 3

Qn5 Is there a cure for HIV/AIDS? 1 2 3

Qn6 Does swallowing of ARV for the HIV-infected person prolong their lives?1 2 3

Qn6a Does abstinence offer full protection from HIV? 1 2 3

Qn6b Does faithfulness offer full protection from HIV? 1 2 3

Qn6a Does correct consistent condom use offer full protection from HIV? 1 2 3

**VMMC PROCEEDURE AND PERCEPTIONS**

*(In this section, select 1 for “True”, 2 for “False”, and 3 for “Don’t know”)*

**I would like to ask you questions on Voluntary Medical Male Circumcision**

True False DK

*Qn7 Circumcision involves removal of one’s foreskin 1 2 3

*Qn11 VMMC differs from traditional circumcision 1 2 3

Qn13 There is no need for an exam before circumcision 1 2 3

*Qn14 An injection is given for pain control before circumcision 1 2 3

Qn15 Circumcision involves removal of the foreskin by a trained

health worker 1 2 3

*Qn16 Tubes are cut during circumcision 1 2 3

**VMMC BENEFITS AND PERCEPTIONS**

True False DK

*Qn17 Circumcision can make a man immune to HIV/AIDS 1 2 3

*Qn18 Circumcision reduces a man’s risk of acquiring HIV 1 2 3

Qn19 Circumcision makes a man more fertile 1 2 3

*Qn20 Circumcision improves genital hygiene 1 2 3

*Qn21 Circumcision makes a man a muslim 1 2 3

*Qn22 Circumcision reduces risk of STIs 1 2 3

*Qn23 Circumcision reduces cervical cancer risk for the female partner 1 2 3

*Qn24 Circumcision reduces risk of HIV transmission to female partner 1 2 3

**VMMC RISKS AND PERCEPTIONS** True False DK

*Qn25 It is normal to bleed after circumcision 1 2 3

Qn26 Excessive pain should be reported to a health worker 1 2 3

Qn27 A man should return immediately in case of swelling after circumcision 1 2 3

Qn28 A man should not be treated for a pus discharge from where he was circumcised

1 2 3

Qn29 Traditional medicine/herbs heal circumcision wounds faster 1 2 3

Qn30 Traditional medicine/herbs can infect the circumcision wound 1 2 3

Qn31 Excess removal of skin during VMMC procedure is normal/recommended

1 2 3

Qn32 Insufficient skin removal during VMMC procedure is not normal/not recommended

1 2 3

Qn33 A man can have reduced desire for sex after circumcision 1 2 3

*Qn34 A man can desire more sexual partners after circumcision 1 2 3

Qn35 Weak erections can happen after circumcision 1 2 3

*Qn36 Sexual performance reduces after circumcision 1 2 3

**GOOD AND BAD PRACTICES AFTER VMMC**

True False Dk

Qn37 A man should return for follow-up after circumcision even if he has no problem 1 2 3

Qn38 It is not necessary to follow health care workers’ instructions after circumcision 1 2 3

Qn39 Self-medicate in case of any problems 1 2 3

Qn40 Apply herbs or medications directly on the wound 1 2 3

Qn41 Do not shower until the wound heals 1 2 3

Qn42 Do not resume sex even if the wound is healed before 6 weeks 1 2 3

Qn43 Avoid heavy duties after circumcision 1 2 3

Qn44 Sutures can be removed by the client 1 2 3

Qn45 Do not wear trousers after circumcision 1 2 3

Qn46 Continue abstinence or faithfulness and condom use after circumcision

1 2 3

Qn47 Involve your sexual partner before and after circumcision 1 2 3

**EXPOSURE TO HIV MESSAGING**

I would like to ask you some questions which will help us know how much you have been exposed to health messages concerning HIV/AIDS. Your responses will be kept confidential

(**Prompted**)

Very likely-1, Somewhat likely-2

Unlikiely- 3, Not at all-4, Don’t know-5

Qn48 How likely are you to have been exposed to HIV

*(In this section, select 1 for a “Yes", 2 for a “No”, and DK for “Do not know”)*

Yes No Dk

Qn49 Do you think VMMC can reduce your risk of acquiring HIV? 1 2 3

Qn50 Have you ever received counseling for HIV/AIDS? 1 2 3

Qn51 Have you ever cared for a person living with HIV? 1 2 3

Qn52 Have you ever been visited by a Village Health Team member? 1 2 3

Qn53 Do you attend community meetings? 1 2 3

Which of the following is your source of health messages? (select all applicable sources)

Qn54a Billboards

Qn54b Health Facilities

Qn54c Radio

Qn54d Television

Qn54e Outreaches

Qn54f Newspapers

Qn54g Friends/Neighbors

Qn54h Other Specify___________

***Thank you for your time.***
