# Supplementary material for: Knowledge on voluntary medical male circumcision in a low uptake setting in northern Uganda
Source: BMC Public Health. 2018 Nov 20;18:1278. doi: 10.1186/s12889-018-6158-2 (PMC6245765; doi:10.1186/s12889-018-6158-2)
Supplement: Supplementary file 2 — Table S1. Comparison of participants with complete responses versus those with incomplete responses to knowledge questions on VMMC. (DOC 45 kb) [file 12889_2018_6158_MOESM2_ESM.doc]

Additional file 2: Table S1: Comparison of participants with complete responses versus those with incomplete responses to knowledge questions on VMMC

|  | **Men** | | | **Women** | | |
| --- | --- | --- | --- | --- | --- | --- |
|  | Complete responses to knowledge questions (N=396) | Incomplete responses to knowledge questions (N=32) | p | Complete responses to knowledge questions (N=50) | Incomplete responses to knowledge questions (N=10) | p |
| **Age**  Mean(SD)  Median(IQR) | 27.2(7.5)  25(22-30) | 25.9(6.2)  25(22-28) | 0.060  0.164 | 28.7(7.2)  29(24-33) | 28.1(8.3)  26(21-32) | 0.779  0.565 |
| **Location, n(%)**  Rural  Urban | 148(45.0)  218(55.0) | 16(50.0)  16(50.0) | 0.581 | 25(50.0)  25(50.0) | 4(40.0)  6(60.0) | 0.732 |
| **Marital status, n(%)**  Married/consensual  Unmarried | 210(53.0)  186(47.0) | 15(46.9)  17(53.1) | 0.502 | 32(64.0)  18(36.0) | 7(70.0)  3(30.0) | 1.000 |
| **Tribe, n(%)**  Acholi  Langi  Other | 343(86.6)  39(9.9)  14(3.5) | 30(9.8)  2(6.3)  0(0.0) | 0.665 | 42(84.0)  2(4.0)  6(12.0) | 7(70.0)  2(20.0)  1(10.0) | 0.202 |
| **Religion, n(%)**  Catholic  Protestant  Other | 273(68.9)  76(19.2)  47(11.9) | 23(71.9)  8(25.0)  1(3.1) | 0.284 | 32(64.0)  13(26.0)  5(10.0) | 5(50.0)  4(40.0)  1(10.0) | 0.670 |
| **Education, n(%)**  None to primary  Secondary  Tertiary/university | 108(27.3)  85(46.7)  103(26.0) | 14(43.8)  3(40.6)  5(15.6) | 0.126 | 27(54.0)  17(34.0)  6(12.0) | 4(40.0)  4(40.0)  2(20.0) | 0.704 |
| **Occupation, n(%)**  Farming  Trading  Student  Other  Unemployed | 02(25.8)  82(20.7)  62(15.7)  04(26.3)  46(11.6) | 9(28.1)  3(9.4)  4(12.5)  10(31.3)  6(18.8) | 0.422 | 12(24.0)  21(42.0)  3(6.0)  9(18.0)  5(10.0) | 3(30.0)  2(20.0)  2(20.0)  2(20.0)  1(10.0) | 0.435 |
| **VMMC status, n(%)***  Uncircumcised  Circumcised | 262(68.7)  124(31.3) | 25(78.1)  7(21.9) | 0.265 | 34(68.0)  16(32.0) | 10(100.0)  0(0.0) | 0.050 |

*For female respondents, VMMC status is that of the male sexual partner
