# Supplementary material for: Knowledge on voluntary medical male circumcision in a low uptake setting in northern Uganda
Source: BMC Public Health. 2018 Nov 20;18:1278. doi: 10.1186/s12889-018-6158-2 (PMC6245765; doi:10.1186/s12889-018-6158-2)
Supplement: Supplementary file 3 — Table S2. Balancing of comparison groups with propensity score weights. Numbers are Standardized mean differences (SMD), and an absolute SMD < 0.1 is desirable for each observed covariate. Only questions with p < 0.05 in bivariate analyses (Table 1) are included. (DOC 50 kb) [file 12889_2018_6158_MOESM3_ESM.doc]

Additional file 3: Table S2: Balancing of comparison groups with propensity score weights. Numbers are Standardized mean differences (SMD), and an absolute SMD <0.1 is desirable for each observed covariate. Only questions with p<0.05 in bivariate analyses (Table 1) are included

|  | **VMMC differs from traditional circumcision** | **Men normally bleed after VMMC** | **Men usually desire more sexual partners after VMMC** | **VMMC reduces sexual performance** | |
| --- | --- | --- | --- | --- | --- |
|  | Male respondents: Fail to accept-Accept | Male respondents: Fail to reject-Reject | Male respondents: Fail to reject-Reject | Male respondents: Fail to reject-Reject | Female respondents: Fail to reject-Reject |
| Age | -0.014 | -0.042 | -0.004 | -0.016 | 0.038 |
| Location: rural | 0.025 | -0.014 | 0.003 | -0.003 | -0.003 |
| Location: urban | -0.025 | 0.014 | -0.003 | 0.003 | 0.003 |
| Marital status: unmarried | 0.002 | -0.061 | -0.011 | 0.002 | 0.059 |
| Marital status: married | -0.002 | 0.061 | 0.011 | -0.002 | -0.059 |
| Tribe: Acholi | 0.023 | 0.007 | -0.021 | -0.006 | -0.022 |
| Tribe: Langi | -0.025 | -0.008 | 0.018 | 0.006 | 0.000 |
| Tribe: Other | 0.000 | 0.000 | 0.010 | -0.000 | 0.024 |
| Religion: Catholic | -0.038 | -0.033 | 0.024 | 0.018 | 0.057 |
| Religion: Protestant | 0.017 | 0.061 | -0.026 | 0.010 | -0.043 |
| Religion: Other | 0.034 | -0.030 | -0.003 | -0.039 | -0.027 |
| Education: None-primary | -0.005 | 0.020 | 0.023 | 0.011 | 0.015 |
| Education: Secondary | -0.016 | -0.007 | -0.020 | -0.019 | 0.022 |
| Education: Tertiary/university | 0.024 | -0.015 | -0.001 | 0.011 | 0.022 |
